# *N*′-[(*E*)-(Furan-2-yl)methyl­idene]-2-[4-(2-methyl­prop­yl)phen­yl]propano­hydrazide

**DOI:** 10.1107/S1600536814003936

**Published:** 2014-02-26

**Authors:** Mehmet Akkurt, Shaaban K. Mohamed, Joel T. Mague, Mustafa R. Albayati, Sabry H. H. Younes

**Affiliations:** a Department of Physics, Faculty of Sciences, Erciyes University, 38039 Kayseri, Turkey; b Chemistry and Environmental Division, Manchester Metropolitan University, Manchester M1 5GD, England; c Chemistry Department, Faculty of Science, Minia University, 61519 El-Minia, Egypt; d Department of Chemistry, Tulane University, New Orleans, LA 70118, USA; e Kirkuk University, College of Science, Department of Chemistry, Kirkuk, Iraq; f Department of Chemistry, Faculty of Science, Sohag University, 82524 Sohag, Egypt

## Abstract

In the title mol­ecule, C_18_H_22_N_2_O_2_, the furan and benzene rings form a dihedral angle of 70.17 (14)°. In the crystal, strong N—H⋯O and weak C—H⋯O hydrogen bonds link the mol­ecules into chains running parallel to [010].

## Related literature   

For the synthesis of compounds of similar structure to Ibuprofen undertaken as part of our ongoing study incorporating non-steroidal anti-inflammatory drugs (NSAIDs) as a substructure in the synthesis of potential bio-active pharmacophors, see: Mohamed *et al.* (2012 ▶, 2013 ▶). For general harmful side-effects of NSAIDs, see: Neeraj *et al.* (2010 ▶); Agrawal *et al.* (2010 ▶); Champion *et al.* (1997 ▶); Asif (2009 ▶). For reduction of these side-effects, see: Parmeshwari *et al.* (2009 ▶); Alert (1958 ▶); Bundgaard (1991 ▶).
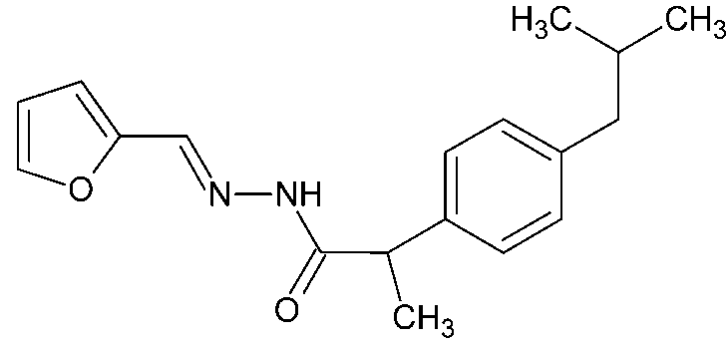


## Experimental   

### 

#### Crystal data  

C_18_H_22_N_2_O_2_
*M* *_r_* = 298.38
Orthorhombic, 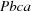

*a* = 11.714 (3) Å
*b* = 8.430 (2) Å
*c* = 33.872 (8) Å
*V* = 3344.8 (14) Å^3^
*Z* = 8
Mo *K*α radiation
μ = 0.08 mm^−1^
*T* = 150 K
0.19 × 0.17 × 0.08 mm

#### Data collection  

Bruker SMART APEX CCD diffractometer
Absorption correction: multi-scan (*SADABS*; Bruker, 2013 ▶) *T*_min_ = 0.52, *T*_max_ = 0.99
55300 measured reflections
4178 independent reflections
2562 reflections with *I* > 2σ(*I*)
*R*_int_ = 0.154

#### Refinement  

*R*[*F* ^2^ > 2σ(*F* ^2^)] = 0.077
*wR*(*F* ^2^) = 0.248
*S* = 1.01
4178 reflections
203 parameters
H atoms treated by a mixture of independent and constrained refinement
Δρ_max_ = 0.37 e Å^−3^
Δρ_min_ = −0.39 e Å^−3^

### 

Data collection: *APEX2* (Bruker, 2013 ▶); cell refinement: *SAINT* (Bruker, 2013 ▶); data reduction: *SAINT*; program(s) used to solve structure: *SHELXS2013* (Sheldrick, 2008 ▶); program(s) used to refine structure: *SHELXL2013* (Sheldrick, 2008 ▶); molecular graphics: *ORTEP-3 for Windows* (Farrugia, 2012 ▶); software used to prepare material for publication: *PLATON* (Spek, 2009 ▶).

## Supplementary Material

Crystal structure: contains datablock(s) global, I. DOI: 10.1107/S1600536814003936/hg5385sup1.cif

Structure factors: contains datablock(s) I. DOI: 10.1107/S1600536814003936/hg5385Isup2.hkl

Supporting information file. DOI: 10.1107/S1600536814003936/hg5385Isup3.cml

CCDC reference: 987953

Additional supporting information:  crystallographic information; 3D view; checkCIF report

## Figures and Tables

**Table 1:** Hydrogen-bond geometry (Å, °)

*D*—H⋯*A*	*D*—H	H⋯*A*	*D*⋯*A*	*D*—H⋯*A*
N2—H2*N*⋯O2^i^	0.96 (3)	1.86 (2)	2.791 (2)	164 (2)
C5—H5⋯O2^i^	0.95	2.49	3.296 (3)	142

Symmetry code: (i) 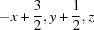.

**Fig. 1.**
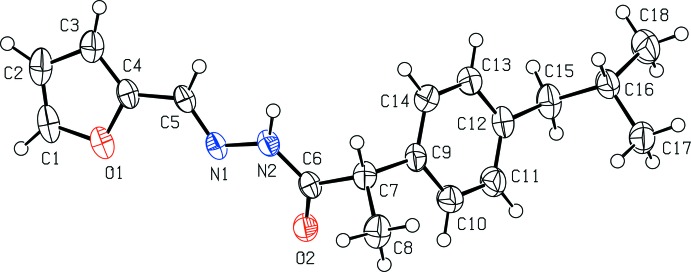
The molecular structure of the title compound (I) with the atom numbering scheme. Displacement ellipsoids for non-H atoms are drawn at the 50% probability level.

**Fig. 2.**
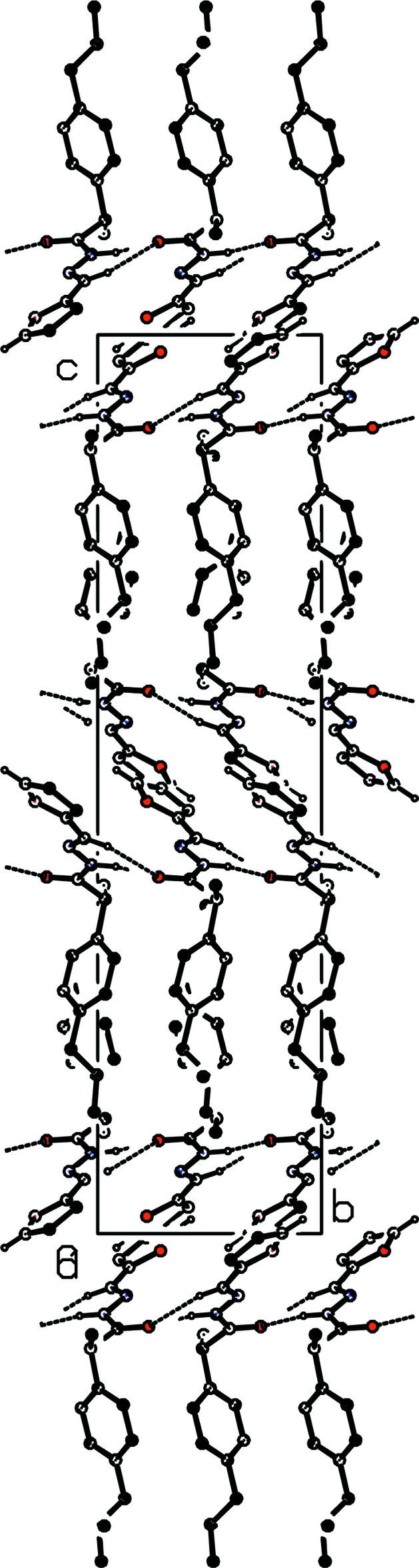
View of the hydrogen bonding and packing of the title compound down the a axis.

## supplementary crystallographic information

### 1. Comment

Ibuprofen, as other common anti-inflammatory drugs (NSAIDs) which are widely employed in the treatment of pain and inflammation, has been reported to be associated with a number of undesirable effects which, in particular, include gastrointestinal (GI) toxicity (Neeraj *et al.*, 2010; Agrawal *et al.*, 2010; Champion *et al.*, 1997). These studies confirmed that gastrointestinal side-effects of Iburofen and other aroylpropanoic acids are due to the presence of a free carboxylic group in the parent drug (Asif, 2009). Therefore, temporary masking or manipulation of the acidic group in NSAID's are promising means to reduce or to abolish the GI toxicity due to the local action mechanism (Parmeshwari *et al.*, 2009; Alert 1958; Bundgaard, 1991). In view of such facts and following to our ongoing study incorporating NSAID's as a substructure in the synthesis of potential bio-active pharmacophors (Mohamed *et al.*, 2012, 2013) we report the crystal structure of the title compound (I).

Fig. 1 shows the title molecule (I). The dihedral angle between the mean planes of the furan ring (O1/C1–C4) and the benzene ring (C9–C14) is 70.17 (14)°. The C5–N1–N2–C6, N2–N1–C5–C4, N1–N2–C6–C7 and N1–N2–C6–O2 torsion angles are -170.46 (19), -176.99 (19), 178.89 (17) and -0.3 (3)°, respectively. The bond lengths and bond angles in (I) are normal.

The crystal packing (Fig. 2) is directed by intermolecular N—H···O and C—H···O hydrogen bonds (Table 1) connecting the molecules into chains running parallel to the *b* axis.

### 2. Experimental

The title compound was prepared according to our reported method (Mohamed *et al*., 2012). Clear orange crystals suitable for X-ray analysis were grown from an ethanol solution of (I). *M*.p. 426–428 K.

### 3. Refinement

The H atoms of N2 was located from a difference Fourier map and refined freely. The other H atoms were placed in geometrically idealized positions and refined using a riding model approximation with *U*_iso_(H) = 1.2 or 1.5*U*_eq_(C).

### Crystal data

**Table:**

C_18_H_22_N_2_O_2_	*F*(000) = 1280
*M**_r_* = 298.38	*D*_x_ = 1.185 Mg m^−^^3^
Orthorhombic, *P**b**c**a*	Mo *K*α radiation, λ = 0.71073 Å
Hall symbol: -P 2ac 2ab	Cell parameters from 9972 reflections
*a* = 11.714 (3) Å	θ = 2.4–28.2°
*b* = 8.430 (2) Å	µ = 0.08 mm^−^^1^
*c* = 33.872 (8) Å	*T* = 150 K
*V* = 3344.8 (14) Å^3^	Slab, clear orange
*Z* = 8	0.19 × 0.17 × 0.08 mm

### Data collection

**Table:**

Bruker SMART APEX CCD diffractometer	4178 independent reflections
Radiation source: fine-focus sealed tube	2562 reflections with *I* > 2σ(*I*)
Graphite monochromator	*R*_int_ = 0.154
Detector resolution: 8.3660 pixels mm^-1^	θ_max_ = 28.4°, θ_min_ = 2.1°
φ and ω scans	*h* = −15→15
Absorption correction: multi-scan (*SADABS*; Bruker, 2013)	*k* = −11→11
*T*_min_ = 0.52, *T*_max_ = 0.99	*l* = −45→45
55300 measured reflections	

### Refinement

**Table:**

Refinement on *F*^2^	0 restraints
Least-squares matrix: full	Hydrogen site location: mixed
*R*[*F*^2^ > 2σ(*F*^2^)] = 0.077	H atoms treated by a mixture of independent and constrained refinement
*wR*(*F*^2^) = 0.248	*w* = 1/[σ^2^(*F*_o_^2^) + (0.1555*P*)^2^ + 0.1359*P*] where *P* = (*F*_o_^2^ + 2*F*_c_^2^)/3
*S* = 1.01	(Δ/σ)_max_ < 0.001
4178 reflections	Δρ_max_ = 0.37 e Å^−^^3^
203 parameters	Δρ_min_ = −0.39 e Å^−^^3^

### Special details

**Table:**

Geometry. Bond distances, angles *etc*. have been calculated using the rounded fractional coordinates. All su's are estimated from the variances of the (full) variance-covariance matrix. The cell e.s.d.'s are taken into account in the estimation of distances, angles and torsion angles
Refinement. Refinement on *F*^2^ for ALL reflections except those flagged by the user for potential systematic errors. Weighted *R*-factors *wR* and all goodnesses of fit *S* are based on *F*^2^, conventional *R*-factors *R* are based on *F*, with *F* set to zero for negative *F*^2^. The observed criterion of *F*^2^ > σ(*F*^2^) is used only for calculating -*R*-factor-obs *etc*. and is not relevant to the choice of reflections for refinement. *R*-factors based on *F*^2^ are statistically about twice as large as those based on *F*, and *R*-factors based on ALL data will be even larger.

### Fractional atomic coordinates and isotropic or equivalent isotropic displacement parameters (Å^2^)

**Table:**

	*x*	*y*	*z*	*U*_iso_*/*U*_eq_	
O1	0.99800 (14)	−0.2805 (2)	0.01925 (5)	0.0464 (6)	
O2	0.64262 (13)	−0.22818 (19)	0.10344 (5)	0.0397 (5)	
N1	0.84096 (15)	−0.1275 (2)	0.06902 (5)	0.0328 (6)	
N2	0.76667 (16)	−0.0292 (2)	0.08961 (5)	0.0330 (6)	
C1	1.0961 (2)	−0.3228 (4)	−0.00048 (8)	0.0540 (10)	
C2	1.1779 (2)	−0.2161 (4)	0.00466 (8)	0.0512 (9)	
C3	1.1314 (2)	−0.0958 (3)	0.02921 (7)	0.0448 (8)	
C4	1.0227 (2)	−0.1395 (3)	0.03736 (6)	0.0360 (7)	
C5	0.93628 (19)	−0.0609 (3)	0.06022 (6)	0.0332 (7)	
C6	0.67103 (19)	−0.0885 (3)	0.10569 (6)	0.0324 (7)	
C7	0.59945 (19)	0.0323 (3)	0.12793 (7)	0.0360 (7)	
C8	0.4770 (2)	0.0268 (4)	0.11260 (8)	0.0483 (9)	
C9	0.6096 (2)	−0.0021 (3)	0.17205 (7)	0.0350 (7)	
C10	0.5416 (2)	−0.1140 (3)	0.19069 (7)	0.0433 (8)	
C11	0.5583 (2)	−0.1527 (3)	0.23008 (7)	0.0450 (8)	
C12	0.6440 (2)	−0.0800 (3)	0.25206 (7)	0.0388 (7)	
C13	0.7101 (2)	0.0338 (3)	0.23343 (7)	0.0435 (8)	
C14	0.6929 (2)	0.0729 (3)	0.19408 (7)	0.0415 (8)	
C15	0.6661 (2)	−0.1200 (3)	0.29488 (7)	0.0468 (9)	
C16	0.6095 (2)	−0.0036 (3)	0.32356 (7)	0.0412 (8)	
C17	0.4816 (2)	−0.0285 (3)	0.32544 (8)	0.0480 (9)	
C18	0.6609 (3)	−0.0161 (5)	0.36474 (9)	0.0651 (13)	
H1	1.10420	−0.41640	−0.01580	0.0650*	
H2	1.25290	−0.21940	−0.00600	0.0610*	
H2N	0.786 (2)	0.081 (3)	0.0917 (7)	0.037 (7)*	
H3	1.16920	−0.00290	0.03820	0.0540*	
H5	0.95010	0.04420	0.06920	0.0400*	
H7	0.63120	0.14040	0.12270	0.0430*	
H8A	0.43080	0.10540	0.12670	0.0720*	
H8B	0.44520	−0.07930	0.11700	0.0720*	
H8C	0.47640	0.05080	0.08430	0.0720*	
H10	0.48250	−0.16510	0.17620	0.0520*	
H11	0.51070	−0.22980	0.24220	0.0540*	
H13	0.76840	0.08630	0.24790	0.0520*	
H14	0.73910	0.15210	0.18210	0.0500*	
H15A	0.74950	−0.12020	0.29960	0.0560*	
H15B	0.63720	−0.22830	0.30030	0.0560*	
H16	0.62380	0.10630	0.31360	0.0490*	
H17A	0.44920	−0.01880	0.29890	0.0720*	
H17B	0.44750	0.05170	0.34270	0.0720*	
H17C	0.46540	−0.13450	0.33590	0.0720*	
H18A	0.74360	0.00020	0.36320	0.0970*	
H18B	0.64510	−0.12160	0.37560	0.0970*	
H18C	0.62710	0.06490	0.38180	0.0970*	

### Atomic displacement parameters (Å^2^)

**Table:**

	*U*^11^	*U*^22^	*U*^33^	*U*^12^	*U*^13^	*U*^23^
O1	0.0561 (11)	0.0513 (11)	0.0318 (9)	0.0040 (8)	0.0054 (7)	−0.0101 (8)
O2	0.0468 (9)	0.0360 (10)	0.0364 (9)	−0.0030 (7)	0.0081 (7)	−0.0032 (7)
N1	0.0397 (10)	0.0379 (11)	0.0209 (9)	0.0040 (8)	0.0003 (7)	−0.0038 (7)
N2	0.0397 (11)	0.0316 (11)	0.0277 (9)	0.0017 (8)	0.0035 (7)	−0.0046 (7)
C1	0.0674 (18)	0.0610 (18)	0.0335 (13)	0.0139 (15)	0.0133 (12)	−0.0083 (12)
C2	0.0536 (15)	0.0672 (19)	0.0327 (13)	0.0138 (14)	0.0121 (11)	0.0043 (12)
C3	0.0488 (14)	0.0530 (15)	0.0326 (13)	0.0032 (12)	0.0078 (10)	0.0013 (11)
C4	0.0460 (13)	0.0406 (13)	0.0213 (10)	0.0040 (10)	0.0015 (9)	0.0011 (9)
C5	0.0415 (12)	0.0381 (13)	0.0199 (10)	0.0033 (10)	−0.0014 (8)	−0.0021 (8)
C6	0.0395 (12)	0.0347 (13)	0.0230 (10)	0.0029 (9)	−0.0005 (8)	0.0003 (8)
C7	0.0403 (12)	0.0346 (12)	0.0332 (12)	0.0030 (10)	0.0062 (9)	0.0006 (9)
C8	0.0463 (15)	0.0582 (17)	0.0403 (14)	0.0085 (12)	0.0037 (11)	0.0045 (12)
C9	0.0401 (13)	0.0350 (12)	0.0300 (11)	0.0033 (9)	0.0082 (9)	−0.0035 (9)
C10	0.0500 (14)	0.0441 (14)	0.0358 (13)	−0.0087 (11)	0.0040 (10)	−0.0021 (10)
C11	0.0578 (15)	0.0421 (14)	0.0351 (13)	−0.0074 (12)	0.0088 (11)	0.0005 (10)
C12	0.0461 (13)	0.0403 (13)	0.0299 (12)	0.0091 (10)	0.0073 (9)	−0.0022 (10)
C13	0.0419 (13)	0.0541 (16)	0.0345 (12)	−0.0024 (11)	0.0032 (10)	−0.0066 (11)
C14	0.0409 (13)	0.0454 (14)	0.0381 (13)	−0.0062 (10)	0.0074 (10)	−0.0017 (10)
C15	0.0509 (15)	0.0574 (17)	0.0320 (13)	0.0134 (12)	0.0038 (10)	0.0020 (11)
C16	0.0483 (14)	0.0474 (15)	0.0280 (12)	−0.0007 (11)	0.0033 (10)	−0.0024 (10)
C17	0.0485 (15)	0.0580 (17)	0.0376 (14)	0.0012 (12)	0.0062 (11)	−0.0019 (11)
C18	0.0608 (18)	0.100 (3)	0.0345 (15)	−0.0053 (17)	−0.0013 (13)	−0.0077 (14)

### Geometric parameters (Å, º)

**Table:**

O1—C1	1.376 (3)	C16—C18	1.523 (4)
O1—C4	1.369 (3)	C1—H1	0.9500
O2—C6	1.226 (3)	C2—H2	0.9500
N1—N2	1.389 (2)	C3—H3	0.9500
N1—C5	1.285 (3)	C5—H5	0.9500
N2—C6	1.342 (3)	C7—H7	1.0000
N2—H2N	0.96 (3)	C8—H8A	0.9800
C1—C2	1.326 (4)	C8—H8B	0.9800
C2—C3	1.420 (4)	C8—H8C	0.9800
C3—C4	1.354 (3)	C10—H10	0.9500
C4—C5	1.437 (3)	C11—H11	0.9500
C6—C7	1.519 (3)	C13—H13	0.9500
C7—C8	1.526 (3)	C14—H14	0.9500
C7—C9	1.527 (3)	C15—H15A	0.9900
C9—C14	1.382 (3)	C15—H15B	0.9900
C9—C10	1.387 (3)	C16—H16	1.0000
C10—C11	1.387 (3)	C17—H17A	0.9800
C11—C12	1.392 (3)	C17—H17B	0.9800
C12—C15	1.511 (3)	C17—H17C	0.9800
C12—C13	1.385 (3)	C18—H18A	0.9800
C13—C14	1.388 (3)	C18—H18B	0.9800
C15—C16	1.532 (3)	C18—H18C	0.9800
C16—C17	1.514 (3)		
			
C1—O1—C4	105.4 (2)	C4—C5—H5	119.00
N2—N1—C5	113.59 (18)	C6—C7—H7	109.00
N1—N2—C6	120.29 (18)	C8—C7—H7	108.00
C6—N2—H2N	121.9 (14)	C9—C7—H7	109.00
N1—N2—H2N	117.8 (14)	C7—C8—H8A	109.00
O1—C1—C2	111.3 (3)	C7—C8—H8B	109.00
C1—C2—C3	106.5 (2)	C7—C8—H8C	109.00
C2—C3—C4	106.6 (2)	H8A—C8—H8B	109.00
O1—C4—C5	119.6 (2)	H8A—C8—H8C	109.00
O1—C4—C3	110.1 (2)	H8B—C8—H8C	110.00
C3—C4—C5	130.3 (2)	C9—C10—H10	119.00
N1—C5—C4	122.4 (2)	C11—C10—H10	119.00
O2—C6—C7	121.7 (2)	C10—C11—H11	120.00
O2—C6—N2	124.0 (2)	C12—C11—H11	120.00
N2—C6—C7	114.4 (2)	C12—C13—H13	119.00
C6—C7—C9	108.36 (19)	C14—C13—H13	119.00
C6—C7—C8	109.3 (2)	C9—C14—H14	120.00
C8—C7—C9	113.6 (2)	C13—C14—H14	120.00
C10—C9—C14	118.1 (2)	C12—C15—H15A	109.00
C7—C9—C14	119.8 (2)	C12—C15—H15B	109.00
C7—C9—C10	122.0 (2)	C16—C15—H15A	109.00
C9—C10—C11	121.1 (2)	C16—C15—H15B	109.00
C10—C11—C12	120.8 (2)	H15A—C15—H15B	108.00
C11—C12—C15	122.6 (2)	C15—C16—H16	108.00
C11—C12—C13	117.7 (2)	C17—C16—H16	108.00
C13—C12—C15	119.7 (2)	C18—C16—H16	108.00
C12—C13—C14	121.4 (2)	C16—C17—H17A	109.00
C9—C14—C13	120.8 (2)	C16—C17—H17B	109.00
C12—C15—C16	113.1 (2)	C16—C17—H17C	109.00
C17—C16—C18	110.1 (2)	H17A—C17—H17B	109.00
C15—C16—C17	111.5 (2)	H17A—C17—H17C	109.00
C15—C16—C18	111.4 (2)	H17B—C17—H17C	110.00
O1—C1—H1	124.00	C16—C18—H18A	109.00
C2—C1—H1	124.00	C16—C18—H18B	109.00
C1—C2—H2	127.00	C16—C18—H18C	109.00
C3—C2—H2	127.00	H18A—C18—H18B	110.00
C2—C3—H3	127.00	H18A—C18—H18C	109.00
C4—C3—H3	127.00	H18B—C18—H18C	110.00
N1—C5—H5	119.00		
			
C1—O1—C4—C5	179.0 (2)	C6—C7—C9—C14	92.0 (3)
C4—O1—C1—C2	0.0 (3)	C8—C7—C9—C10	37.3 (3)
C1—O1—C4—C3	0.1 (3)	C8—C7—C9—C14	−146.4 (2)
C5—N1—N2—C6	−170.46 (19)	C7—C9—C10—C11	174.8 (2)
N2—N1—C5—C4	−176.99 (19)	C14—C9—C10—C11	−1.6 (4)
N1—N2—C6—C7	178.89 (17)	C7—C9—C14—C13	−174.5 (2)
N1—N2—C6—O2	−0.3 (3)	C10—C9—C14—C13	1.9 (4)
O1—C1—C2—C3	−0.1 (3)	C9—C10—C11—C12	0.0 (4)
C1—C2—C3—C4	0.1 (3)	C10—C11—C12—C13	1.2 (4)
C2—C3—C4—O1	−0.1 (3)	C10—C11—C12—C15	−179.2 (2)
C2—C3—C4—C5	−178.9 (2)	C11—C12—C13—C14	−0.9 (4)
O1—C4—C5—N1	9.8 (3)	C15—C12—C13—C14	179.5 (2)
C3—C4—C5—N1	−171.5 (2)	C11—C12—C15—C16	−97.3 (3)
O2—C6—C7—C8	−53.5 (3)	C13—C12—C15—C16	82.3 (3)
O2—C6—C7—C9	70.8 (3)	C12—C13—C14—C9	−0.7 (4)
N2—C6—C7—C8	127.4 (2)	C12—C15—C16—C17	73.6 (3)
N2—C6—C7—C9	−108.4 (2)	C12—C15—C16—C18	−163.0 (2)
C6—C7—C9—C10	−84.3 (3)		

### Hydrogen-bond geometry (Å, º)

**Table:**

*D*—H···*A*	*D*—H	H···*A*	*D*···*A*	*D*—H···*A*
N2—H2*N*···O2^i^	0.96 (3)	1.86 (2)	2.791 (2)	164 (2)
C5—H5···O2^i^	0.95	2.49	3.296 (3)	142

Symmetry code: (i) −*x*+3/2, *y*+1/2, *z*.
